# Low-Density Lipoprotein Internalization, Degradation and Receptor Recycling Along Membrane Contact Sites

**DOI:** 10.3389/fcell.2022.826379

**Published:** 2022-01-24

**Authors:** Mohammad Majharul Islam, Iryna Hlushchenko, Simon G. Pfisterer

**Affiliations:** Department of Anatomy, Faculty of Medicine, University of Helsinki, Helsinki, Finland

**Keywords:** low-density lipoprotein receptor (LDLR), low-density lipoprotein (LDL), hypercholesterolemia, membrane contact site, endosomal recycling, endosomal degradation

## Abstract

Low-density lipoprotein (LDL) internalization, degradation, and receptor recycling is a fundamental process underlying hypercholesterolemia, a high blood cholesterol concentration, affecting more than 40% of the western population. Membrane contact sites influence endosomal dynamics, plasma membrane lipid composition, and cellular cholesterol distribution. However, if we focus on LDL-related trafficking events we mostly discuss them in an isolated fashion, without cellular context. It is our goal to change this perspective and to highlight that all steps from LDL internalization to receptor recycling are likely associated with dynamic membrane contact sites in which endosomes engage with the endoplasmic reticulum and other organelles.

## Introduction

Lipoproteins are transport shuttles in the circulation, delivering cholesterol to different destinations. The balance of lipoprotein production and clearance determines a person’s blood cholesterol level. Accumulation of cholesterol-enriched low-density lipoprotein (LDL) is a hallmark of hypercholesterolemia, the main risk factor for cardiovascular disease (CVD), one of the most common causes of death worldwide (Mach et al., 2019; Borén et al., 2020).

Here we focus on how cells take up LDL in a regulated process mediated by the LDL receptor (LDLR). Patients with a homozygous mutation in *LDLR* can display more than five-fold higher LDL levels and experience severe cardiovascular complications before adolescence. Also, heterozygous carriers of *LDLR* mutations are at severe CVD risk, experiencing drastically elevated LDL concentrations (Cuchel et al., 2014). Interestingly, genetic defects in LDLR and proteins associated with LDLR trafficking predispose to a greater CVD risk, even when compared to individuals with similar blood cholesterol levels (Trinder et al., 2020). Probably, this is due to life-long exposure to elevated LDL levels or longer residence time of LDL particles in the bloodstream. This highlights the relevance of cellular LDL internalization in the development of hypercholesterolemia and CVD, and the importance of elucidating additional aspects of this pathway.

**FIGURE 1:**
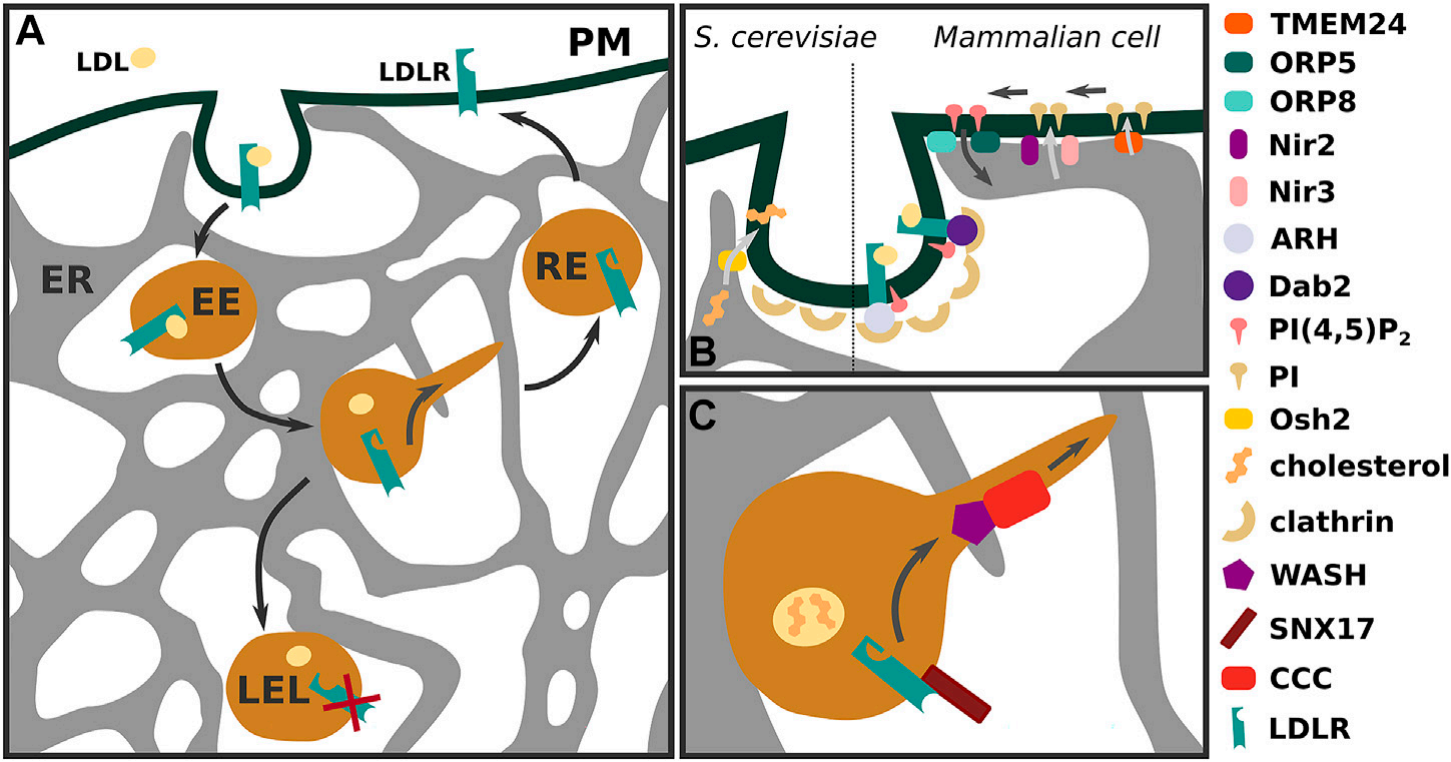
LDLR trafficking in an interconnected membrane system **(A)** Low-density lipoprotein (LDL) receptor (LDLR) bound to its cargo is internalised via clathrin-mediated endocytosis. Within early endosomes (EE) LDLR separates from LDL and is sorted to recycling endosomes (RE) for transport to the plasma membrane. Receptors that fail to separate from LDL or are marked for degradation by extracellular or intracellular factors remain in the maturing endosomal system, resulting in their degradation in late endosomes and lysosomes (LELs). **(B)** Membrane contact sites between endoplasmic reticulum (ER) and plasma membrane are lipid exchange routes in both *S. cerevisiae* and mammalian cells, affecting sterol and PI(4,5)P_2_ abundance. In *S. cerevisiae* sterol transfer to the budding vesicle is important for fission. In mammalian cells PI(4,5)P_2_ is shuffled between PM and ER via ORP5 and ORP8 proteins and PM PI(4,5)P_2_ pools can be replenished by PI transfer via Nir2, Nir3 and TMEM24. **(C)** Sorting of LDLR is enhanced by sorting nexin 17 (SNX17) and requires the WASH and COMMD/CCDC22/CCDC93 (CCC) complex for efficient recycling.

LDL binds to LDLR on the cell surface and is internalized via clathrin-mediated endocytosis (Brown and Goldstein, 1979). In the acidic environment of the early endosome, LDL dissociates from LDLR. Whilst a majority of LDLR is recycled back to the plasma membrane, LDL remains in the maturing endosomal system, resulting in degradation in late endosomes and lysosomes (LELs) (Figure 1A **)** (Brown and Goldstein, 1986). LDLR can join the path to degradation when it does not dissociate from LDL (Davis et al., 1987) or when it is specifically targeted by proprotein convertase subtilisin/kexin type 9 (PCSK9) (Zhang et al., 2007) or inducible degrader of LDLR (IDOL) (Zelcer et al., 2009).

Efficient cholesterol export from LELs and transport to the endoplasmic reticulum (ER) plays an important role in regulating cholesterol synthesis and LDLR expression. Low cholesterol concentrations in the ER result in nuclear translocation of the transcription factor sterol regulatory element-binding protein 2 (SREBP-2) and activation of cholesterol synthesis and LDLR expression. On the other hand, an elevated cholesterol concentration in the ER results in SREBP-2 retention in the ER, reduced LDLR expression and lower LDL uptake. Consequently, defective cholesterol egress from LELs and reduced transport to the ER leads to higher LDL internalization rates (Ikonen 2008; Luo et al., 2020).

The majority of endosomes are in close contact with the ER and membrane contacts between both organelles influence endosomal function as well as fission of recycling vesicles from early endosomes (Eden et al., 2016; Rowland et al., 2014) (Figure 1A). Furthermore, membrane contact sites between the ER and endosomes, Golgi or the plasma membrane facilitate intracellular cholesterol transport, controlling cellular cholesterol balance and influencing transcriptional regulation of LDLR (Wilhelm et al., 2017; Mesmin et al., 2013b; Sandhu et al., 2018). Here we want to highlight those contact sites that appear relevant for LDL internalization and degradation, or enable an efficient retrieval of LDLR to the plasma membrane.

### LDL-LDLR Internalization in Light of Membrane Contact Sites

For efficient internalization of the LDL-LDLR complex from the plasma membrane, LDLR is clustered into coated pits (Anderson et al., 1977; Anderson et al., 1982). The initiation, growth, and maturation of coated pits and vesicles is a tightly regulated process dependent on the plasma membrane levels of phosphatidylinositol-(4,5)-bisphosphate (PI(4,5)P_2_) (Antonescu et al., 2011). Internalization of the LDL-LDLR complex starts with the binding of the adaptor proteins ARH (autosomal recessive hypercholesterolemia) or Dab2 (disabled-2) to the LDLR cytoplasmic tail (Sirinian et al., 2005; Maurer and Cooper, 2006). Adaptor protein interaction with LDLR and PI(4,5)P_2_ is crucial for the formation of clathrin-coated pits, and recruitment of accessory proteins such as AP2 and clathrin stimulate clathrin-coated vesicle generation (Figure 1B **)** (Mettlen et al., 2010).

There are two main routes how membrane contact sites could contribute to the regulation of LDLR internalization. 1) Via influencing the lipid and PI(4,5)P_2_ composition of the plasma membrane. 2) Through direct connection with the nascent endosome, regulating the vesicle formation process.

Several mechanisms have been proposed on how membrane contact sites can influence plasma membrane PI(4,5)P_2_ levels. Oxysterol-binding protein (OSBP)-related proteins (ORP) 5 and 8 localize to plasma membrane-ER (PM-ER) contact sites dependent on PI(4,5)P_2_ and appear to transport phosphatidylserine to the plasma membrane in exchange for PI(4,5)P_2_, with ORP5/ORP8 depletion resulting in PM accumulation of PI(4,5)P_2_ (Ghai et al., 2017) (Figure 1B). In an alternative mechanism, ORP5/ORP8 plasma membrane localization is influenced by both PI4P and PI(4,5)P_2_ and in this case plasma membrane PI(4,5)P_2_ levels are modulated by PI4P transport at PM-ER contacts (Sohn et al., 2018). Overall, PI(4,5)P_2_ formation is limited by the amount of available PI4P and PI precursors, of which PI is synthesized at the ER (Kim et al., 2015; Chang and Liou, 2015; Chang et al., 2013). Nir3 localizes to PM-ER contact sites and maintains a basal PI pool at the plasma membrane from which PI(4,5)P_2_ can be generated via PI 4-kinase and PI4P 5-kinase (Chang and Liou, 2015). Activation of signaling receptors can lead to a rapid local PI(4,5)P_2_ depletion through stimulation of phospholipase C (PLC). This evokes Nir2 translocation to PM-ER contact sites and rapid transfer of PI from the ER to the PM in exchange for phosphatidic acid resulting in PI(4,5)P_2_ reformation (Figure 1B) (Chang and Liou, 2015; Kim et al., 2015). Furthermore, transmembrane protein 24 (TMEM24) can mediate plasma membrane PI replenishment at PM-ER contact sites, resulting in PI4,5P_2_ reformation during glucose-stimulated signaling (Lees et al., 2017) (Figure 1B **)**.

Therefore, even though we lack direct support for this hypothesis, it appears likely that membrane contact sites influence clathrin-mediated endocytosis in a localized fashion through the modulation of PI(4,5)P_2_ abundance, acting together with lipid kinases and phosphatases (Posor et al., 2015).

Interestingly, in *S. cerevisiae* membrane contact sites between the ER and the forming endosome have been observed. This involves the yeast ORP protein Osh2 and results in actin recruitment and vesicle fission (Figure 1B **)** (Encinar del Dedo et al., 2017). Furthermore, Osh2 is involved in mediating sterol transport at these contact sites, which appears to be important for endocytosis when plasma sterol availability is limited (Encinar del Dedo et al., 2021). Also in plants PM-ER contact sites can influence endocytosis. Plant VAP (Vesicle-Associated Membrane Protein-Associated Protein) proteins (VAP27-1 and VAP27-3) mediate contact formation through interaction with PIPs and clathrin at endocytic membranes, facilitating endocytosis (Stefano et al., 2018). This highlights multiple options of how membrane contact sites could influence clathrin-mediated endocytosis and internalization of the LDL-LDLR complex.

Soon after the clathrin-coated vesicle detaches from the plasma membrane, the coat proteins disassemble, PI(4,5)P_2_ is hydrolyzed and the vesicles merge into the early endosomal system (Kaksonen and Roux, 2018). Around 80% of early endosomes are in contact with the ER (Friedman et al., 2013), indicating that this is also the case for those containing the LDL-LDLR complex. At this stage, separate trafficking routes emerge, LDLR can be sorted into recycling endosomes, whilst LDL and some LDLRs are staying on a path to degradation in late endosomes and lysosomes (Wijers et al., 2015).

### LDLR Recycling in an Interconnected Endosome-ER Meshwork

LDLR recycling is activated once the ligand and receptor dissociate in the early endosomal system. At this stage, a conformational change of the LDLR impedes its degradation and makes it available for recycling (Davis et al., 1987; Surdo et al., 2011). Possibly, sorting nexins (SNXs) play an important role in redirecting LDLR towards the plasma membrane. SNX17 binds to the LDLR cytoplasmic tail and SNX17 overexpression increases the LDL internalization rate (Stockinger, 2002; Burden et al., 2004), suggesting a role in LDLR recycling. However, we lack loss-of-function information to say that LDLR sorting depends on SNX17. Therefore, it is possible that other proteins can initiate LDLR recycling as well.

We know that efficient LDLR recycling requires an intact Wiskott–Aldrich syndrome protein and SCAR homolog (WASH) and COMMD/CCDC22/CCDC93 (CCC) complex (Figure 1C) (Bartuzi et al. 2016; Wijers et al., 2019; Rimbert et al., 2020). The WASH components WASH1 and FAM21 co-precipitate with LDLR and WASH1 deficient cells show increased LDLR degradation, reduced surface expression, and LDL uptake. Defects in LDLR recycling in WASH1 deficient cells can be rescued by re-expressing a wild type but not a WASH1 mutant, which fails to initiate F-actin polymerization via Arp2/3 activation (Bartuzi et al., 2016). WASH-mediated actin polymerization plays a pivotal role in endosome fission from the sorting endosome (Derivery et al., 2009). 80% of endosomal tubules undergo fission at an intersection point with the ER. Interestingly, FAM21 localizes to the neck of endosomal tubules and nearly all of these sites overlap with the ER (Rowland et al., 2014). Moreover, WASH activity is regulated by endosomal PI(4)P levels which in turn are influenced by OSBP (Oxysterol Binding Protein) and VAP acting at endosome-ER contact sites (Dong et al., 2016). Tight control of endosomal PI(4)P levels appears important in endosomal fission as OSBP inactivation leads to PI(4)P accumulation and exaggerated actin polymerization (Dong et al., 2016). On the other hand, PI(4)P is coupled to phosphatidylserine delivery to the endosome from the ER *via* ORP10. A defect in this process also impairs effective retrograde trafficking of endosomal cargo and endosomal fission (Kawasaki et al., 2021).

Even though endosomal fission has mostly been studied in connection with endosomal sorting towards the Golgi (Rowland et al., 2014; Dong et al., 2016; Hoyer et al., 2018), it is fair to speculate that ER/endosome interconnections are important for LDLR recycling towards the plasma membrane as well.

### LDL Degradation: Holding on to the ER Whilst Reaching out to Other Organelles

**FIGURE 2:**
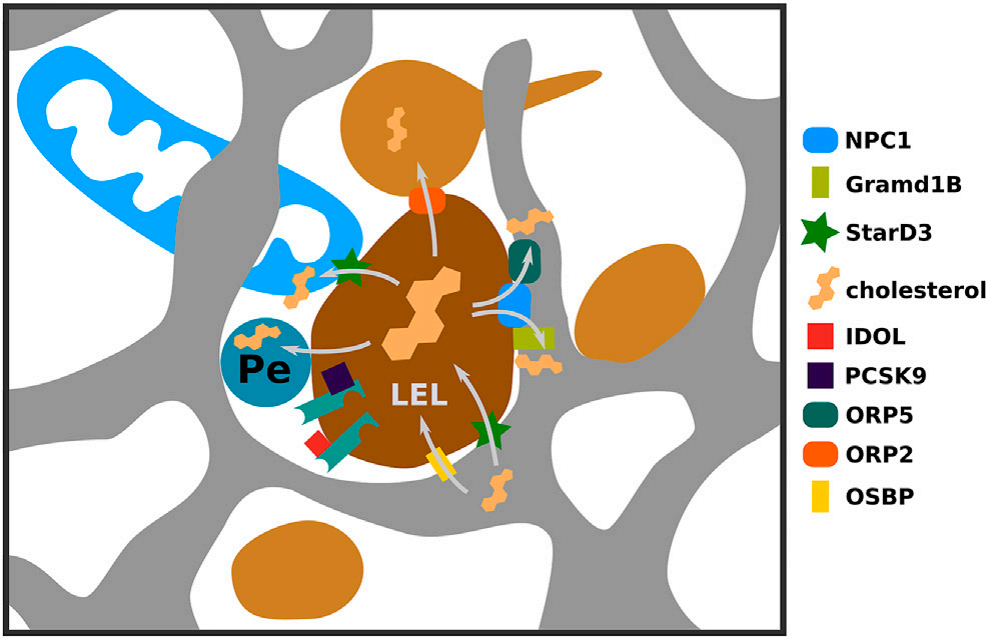
Multiple routes of endosomal cholesterol transport via membrane contact sites. LDL-derived cholesterol is liberated in late endosomes and lysosomes (LEL) and is made available to other cellular compartments via different mechanisms. Here we highlight several contact sites involved in this process. NPC1 via interaction with ORP5 or GramD1B can stimulate contacts between LELs and ER mediating cholesterol efflux to the ER. If NPC1 mediated export to the ER is impaired, cholesterol can be transferred to mitochondria via contacts established via StarD3. Also LELs can engage in contacts with peroxisomes and recycling endosomes for cholesterol export and cholesterol can be transported in reverse direction from ER to LEL.

LDL embarks on a path to degradation in late endosomes/lysosomes upon dissociating from LDLR. LDLR can join this path if targeted by PCSK9, IDOL, or upon failure to dissociate from LDL. On this path endosome association with the ER increases to nearly 100% (Friedman et al., 2013). This close association appears to play a key role in exporting LDL-derived cholesterol from endosomes to the ER (Figure 2 **)**. Whilst there are multiple pathways for cholesterol export from endosomes (Kanerva et al., 2013; Takahashi et al., 2021) it has been shown that 30% of endosomal cholesterol is transported directly to the ER (Neufeld et al., 1996). Lysosomal acid lipase liberates LDL-derived cholesterol in the endosomal lumen (Chang et al., 2006). Then cholesterol gets inserted into the LEL limiting membrane through the concerted action of Nieman Pick Type C 2 (NPC2) and NPC1 proteins (Infante et al., 2008).

The first evidence for the involvement of membrane contact sites in redistributing LDL-derived cholesterol came from studies involving oxysterol binding protein (OSBP) related protein (ORP) 5 (ORP5) (Du et al., 2011). ORP5, an ER-resident protein, interacts with NPC1 in the LEL limiting membrane, establishing a connection between both organelles. Upon LDL load, depletion of ORP5 leads to cholesterol accumulation in LEL membranes (Du et al., 2011). Recent findings further strengthen that protein-protein interactions with NPC1 facilitate LEL-ER contact site formation and cholesterol transport towards the ER (Höglinger et al., 2019). NPC1 depletion reduces ER-lysosome contacts, whilst NPC1 overexpression increases them. Moreover, NPC1 interacts with Gramd1B/AsterB, a novel contact site protein previously implied in PM to ER cholesterol transport (Sandhu et al., 2018; Höglinger et al., 2019). Similar to NPC1, Gramd1B influences LEL-ER contact site formation, and Gramd1B depletion results in endosomal cholesterol accumulation. Interestingly, LEL-ER contact site restoration stimulates cholesterol export even without NPC1 (Höglinger et al., 2019) suggesting that either close proximity itself can lead to cholesterol transport, or that other proteins mediate transport. One such protein could be ORP1L, which localizes to LELs and influences LEL-ER contact site formation (Rocha et al., 2009). Deficiency of ORP1L leads to cholesterol accumulation in LELs and reduced transport towards the ER (Zhao and Ridgway, 2017). Whilst this can indicate that ORP1L affects transport of LDL derived cholesterol along LEL-ER contact sites, this could also happen via more indirect means of transport.

Besides LEL-ER contact sites, LELs engage in membrane contacts with multiple organelles to ensure efficient cholesterol delivery within cells. Contacts between LELs and recycling endosomes facilitate cholesterol transport towards the plasma membrane (Takahashi et al., 2021) and also LEL-Peroxisome contacts can stimulate LEL cholesterol export (Figure 2) (Chu et al., 2015). Moreover, defective ER-Lysosome contacts are compensated by increased LEL-mitochondria contacts, resulting in increased cholesterol transport towards mitochondria, a process which is dependent on the StarD3 protein (Charman et al., 2010; Höglinger et al., 2019). This is a striking effect, especially as StarD3 itself is involved in mediating ER-LEL contacts in cholesterol-depleted conditions to deliver newly synthesized cholesterol towards endosomes (Wilhelm et al., 2017). Reverse cholesterol transport from the ER to endosomes is important for efficient multivesicular body formation as blocking this step results in defective degradation of signaling receptors when access to LDL-cholesterol is limited (Eden et al., 2016). Furthermore, reverse cholesterol transport at LEL-ER contact sites, mediated by OSBP influences mTOR recruitment and activation at LELs, contributing to the regulation of autophagy (Lim et al., 2019).

There are many open questions regarding LDL-cholesterol redistribution via LEL-ER contact sites. These involve the spatio-temporal involvement of proteins in multiple contact sites with different organelles (Gramd1B (Sandhu et al., 2018; Höglinger et al., 2019; Naito et al., 2019; Ferrari et al., 2020; Ercan et al., 2021), ORP5 (Sohn et al., 2018; Du et al., 2011, 2019), ORP1L (Rocha et al., 2009; Boutry and Kim 2021), OSBP (Mesmin et al., 2013a; Lim et al., 2019) and StarD3 (Wilhelm et al., 2017; Höglinger et al., 2019)), and influence of the cellular cholesterol distribution on the formation of LEL-ER contact sites by different players (Rocha et al., 2009; Höglinger et al., 2019; Lim et al., 2019).

## Conclusion

Multiple different membrane contact sites can converge with the LDL internalization and degradation path. Whilst most reliable data exists for the involvement of contact sites in transporting cholesterol between LELs and the ER, we can only extrapolate that membrane contact sites also influence LDL internalization and LDLR recycling events. We believe that more emphasis should be directed to elucidate how membrane contact sites influence clathrin-mediated endocytosis and LDLR trafficking.
